# Gankyrin sustains PI3K/GSK-3β/β-catenin signal activation and promotes colorectal cancer aggressiveness and progression

**DOI:** 10.18632/oncotarget.13215

**Published:** 2016-11-08

**Authors:** Feng He, Huacui Chen, Ping Yang, Qianlong Wu, Tong Zhang, Chengxing Wang, Jianchang Wei, Zhuanpeng Chen, He Hu, Wanglin Li, Jie Cao

**Affiliations:** ^1^ Department of General Surgery, Guangzhou Digestive Disease Center, Guangzhou First People's Hospital, Guangzhou Medical University, Guangzhou 510180, China; ^2^ Department of Pathology, Interdepartmental Program in Vascular Biology and Therapeutics, Yale University School of Medicine, New Haven, Connecticut 06520, USA

**Keywords:** gankyrin, colorectal cancer, metastasis, PI3K/Akt signaling, Wnt/β-catenin signaling

## Abstract

High levels of angiogenesis, metastasis and chemoresistance are major clinical features of colorectal cancer (CRC), a lethal disease with a high incidence worldwide. Aberrant activation of Wnt/β-catenin pathway contributes to CRC progression. However, little is known about regulatory mechanisms of the β-catenin activity in cancer progression. Here we report that Gankyrin was markedly upregulated in primary tumor tissues from CRC patients and was associated with poor survival. Moreover, we demonstrated that overexpressing Gankyrin promoted, while knockdown of Gankyrin impaired, the aggressive phenotype of proliferation, angiogenesis, chemoresistance and metastasis of CRC cells both *in vitro* and *in vivo*. Importantly, we found a unique molecular mechanism of Gankyrin in CRC cells signaling transduction, that regulated the cross-talk between PI3K/Akt and Wnt/β-catenin signaling pathways, sustaining PI3K/GSK-3β/β-catenin signal activation in CRC. Therefore, these findings not only reveal a mechanism that promotes aggressiveness and progression in CRC, but also provide insight into novel molecular targets for antitumor therapy in CRCs.

## INTRODUCTION

Colorectal cancer (CRC) is one of the most prevalent cancers and leading cancer-related mortality worldwide [1]. Distant metastasis is the main cause of death in patients with CRC. Although surgical resection combined with adjuvant therapy is efficient at the early stages of disease, subsequent relapse and metastasis often occur [2, 3]. Angiogenesis is necessary for CRC growth and metastasis [4]. Additionally, CRC cells proliferate rapidly and are highly resistant to apoptosis, resulting in significant insensitivity to chemotherapy [5, 6]. Therefore, investigation of the mechanisms that contribute to metastasis, angiogenesis and chemoresistance may help to further uncover the biological basis of CRC progression and improve clinical therapy [7–9].

Wnt signaling has an essential role in various diseases, and aberrant activation of Wnt/β-catenin signaling is important reason for the occurrence and development in CRC [10, 11]. Mutations in Wnt/β-catenin pathway components including APC, GSK-3β, and β-catenin itself are well-established causes of aberrant signaling activation leading to cancer [12, 13]. Nuclear β-catenin interacts with T cell factor/lymphoid enhancer factor (LEF/TCF) family of transcription factors to induce the expression of target genes such as Cyclin D1 and Myc that can lead to cancer progression [14, 15]. The fact that blockade of Wnt/β-catenin signaling in CRC both *in vitro* and *in vivo* has propelled intensive efforts to develop therapeutic strategies that target this pathway [16, 17].

Gankyrin (also known as PSMD10), was originally identified as a regulatory subunit of the 26S proteasome complex [18]. Research showed that Gankyrin was expressed in almost all eukaryotic cells, especially in many kinds of cancers, such as hepatocellular carcinoma (HCC) [19, 20], CRC [21, 22], esophageal squamous cell carcinoma (ESCC) [23], lung cancers [24], and breast cancer [25]. Gankyrin acts as oncogene, binds to CDK4 and the tumor suppressor retinoblastoma protein (Rb), and accelerates phosphorylation and proteasomal degradation of RB, thus leading to cell cycle progression [26]. Gankyrin has an anti-apoptotic activity, by binding to MDM2, a major E3 ubiquitin ligase for p53, and increases ubiquitylation and degradation of p53 [27]. Gankyrin is responsible for C/EBP protein elimination, leading to promote steps of carcinogenesis [28]. Additionally, Gankyrin mediates Ras-induced tumorigenesis by suppressing ROCK activity [24]. Although Gankyrin has been proven to be involved in carcinogenesis and progression in various cancers, whether it contributes to cancer metastasis, angiogenesis and resistance to chemotherapy in CRC, still remains unclear.

In the present study, we report Gankyrin as an essential regulator of CRC aggressiveness and progression by sustaining PI3K/GSK-3β/β-catenin signal activation by way of mediating the cross-talk between PI3K/Akt and Wnt/β-catenin canonical signaling pathways. This study reveals a novel mechanism that may contribute to the poor prognosis of CRC and strongly highlight the potential of a therapeutic target in patients with CRC.

## RESULTS

### Increased expression of Gankyrin correlates with CRC progression and poor prognosis

**Figure 1:**
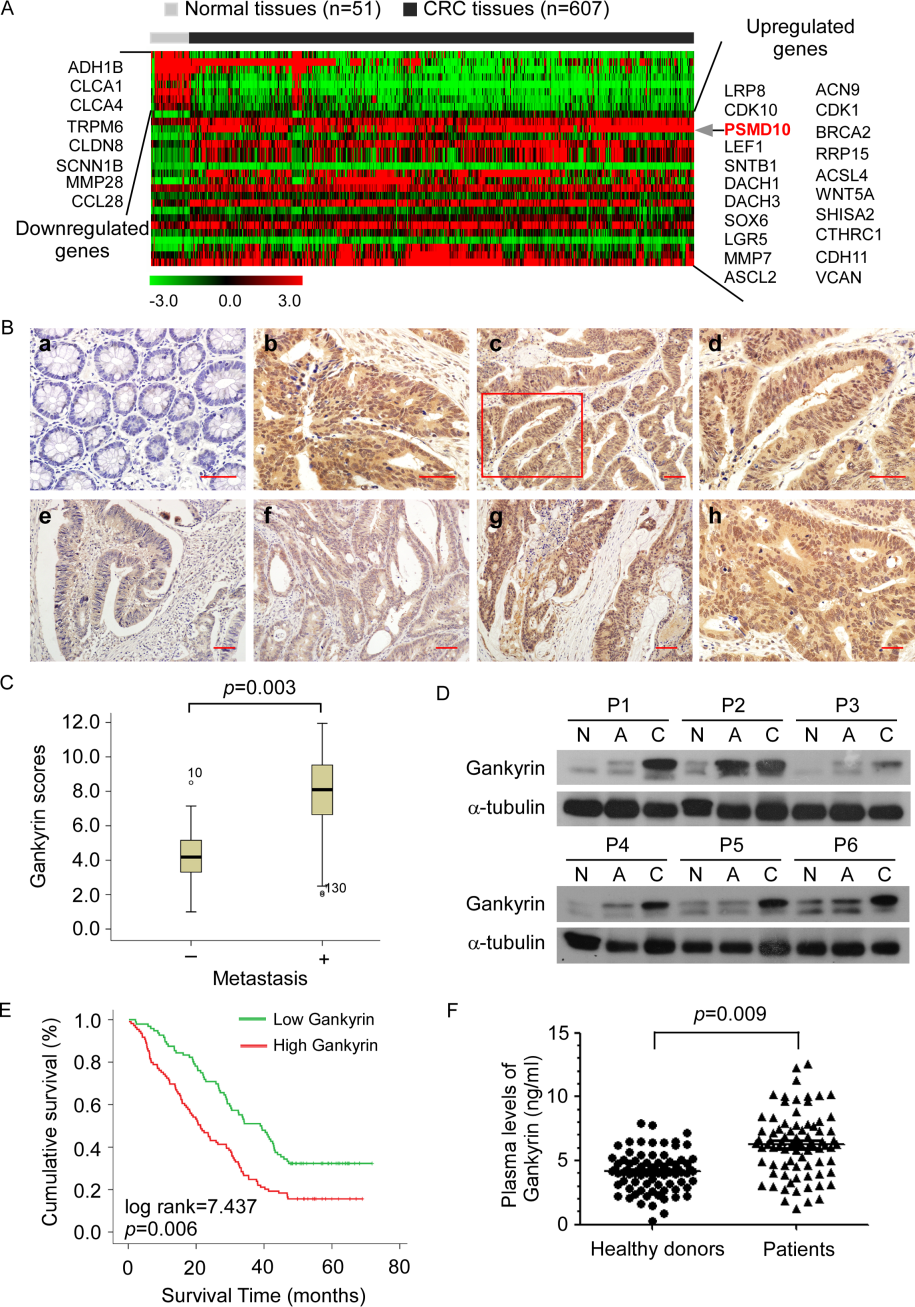
Increased expression of Gankyrin correlates with colorectal cancer (CRC) progression and poor prognosis (**A**) Heat map diagram showing the differentially expressed genes from TCGA database of 607 primary CRC tissues and 51 normal colorectal tissues (Limma moderated *t*-test *P* < 0.05, fold change cut off > 1.5 or < 0.66). The color scale represents the expression level of a gene above (red), below (green), or at the mean expression level (black) across all samples. (**B**) Immunohistochemistry (IHC) staining of Gankyrin expression in normal colorectal tissues (a) and human CRC tissues (b). The expression and localization of Gankyrin in tumor cells (c). The boxed area in (c) was enlarged and shown in (d). Gankyrin expression in tumor cells of CRC tissues from patients with clinical stages I-IV (e-h). Scale bars, 50 μm. (**C**) Gankyrin expression scores between patients with (*n* = 62) and without (*n* = 145) CRC liver metastases are shown as box plots, with the horizontal lines representing the median; the bottom and top of the boxes representing the 25th and 75th percentiles, respectively; and the vertical bars representing the range of data. (**D**) Western blotting analysis of Gankyrin expression in normal colorectal tissues (N), the adjacent noncancerous tissues (A) and paired primary CRC tissues (C). (**E**) Kaplan-Meier overall survival curves for patients with CRC stratified by low (*n* = 108) and high (*n* = 109) expression of Gankyrin (*P* = 0.009). (**F**) ELISA analysis of the plasma levels of Gankyrin in 70 CRC patients and 80 age-matched healthy controls.

By analyzing THE CANCER GENOME ATLAS (TCGA) database obtained from 607 primary CRC tissues and 51 normal colorectal tissues, Gankyrin mRNA was identified to be significantly upregulated in CRC tissues compared with normal tissues (Figure 1A, Supplementary Figure S1). To investigate the role of Gankyrin in the progression of CRC, we first examined the expression of Gankyrin in human CRC tissues. Immunohistochemical (IHC) analysis was conducted to study Gankyrin protein expression in 217 human paraffin-embedded CRC samples. The data showed that the Gankyrin expression was markedly upregulated in CRC tissues compared to the paired adjacent noncancerous tissues (Figure 1B). In addition, intense and high coverage of cell cytoplasm and nucleus expression of Gankyrin was observed in CRC tissues of clinical stages III-IV. Importantly, Gankyrin expression was markedly increased in CRC tissues with liver metastases compared with those without liver metastases (Figure 1C). Likewise, western blotting analysis showed that Gankyrin protein expression was significantly increased in CRC tissues compared to normal colorectal tissues and matched adjacent normal tissues (Figure 1D). Collectively, these results demonstrate that Gankyrin expression is highly elevated in CRC tissues.

To evaluate whether upregulation of Gankyrin correlates with clinical CRC progression, we examined Gankyrin expression in 217 human CRC specimens. Statistical analysis revealed that Gankyrin expression significantly correlated with clinical stage (*P* = 0.003), T classification (*P* = 0.004), N classification (*P* < 0.001) and M classification (*P* = 0.015) in patients with CRC (Table 1). Importantly, statistical analysis revealed that high Gankyrin levels inversely correlated with shorter overall survival of patients with CRC (*P* = 0.006) (Figure 1E). Univariate and multivariate analysis revealed that N classification, M classification and Gankyrin expression were each recognized as independent prognostic factors in CRCs (Table 2). Intriguingly, the mean plasma level of Gankyrin was ~1.5 fold higher in the CRC patients (6.48 ng/ml) as compared with that in the healthy controls (4.16 ng/ml) (Figure 1F). Taken together, these results suggest a possible link between increased expression of Gankyrin and the progression of human CRCs.

**Table 1:** Association between Gankyrin expression in tissue and clinicopathological characteristics of 217 CRC patients

Patient characteristics	Number	Gankyrin expression				*P* value
		Low (%)		High (%)		
Gender						
Male	118	54	45.7%	64	54.3%	0.466
Female	99	43	43.4%	56	56.6%	
Age (years)						
≥ 60	135	60	44.5%	75	55.5%	0.928
< 60	82	37	45.1%	45	54.9%	
Histological differentiation						
Well	54	30	55.6%	24	44.4%	0.139
Moderate	105	44	41.9%	61	58.1%	
Poorly	58	23	39.7%	35	60.3%	
Clinical stage						
Early stage (stage I-II)	126	65	51.5%	61	48.4%	0.003
Late stage (stage III-IV)	91	32	35.2%	59	64.8%	
T classification						
T1	32	22	66.7%	10	30.3%	0.004
T2	62	28	45.2%	34	54.8%	
T3	110	43	39.1%	67	60.9%	
T4	13	5	38.5%	8	61.5%	
N classification						
N0	112	64	57.1%	48	42.9%	< 0.001
N1-N2	105	32	30.5%	73	69.5%	
M classification						
No	146	77	52.7%	69	47.3%	0.015
Yes	71	23	32.4%	48	67.6%	

**Table 2:** Univariate and multivariate analysis Cox regression analysis of 5-year overall survival in CRC patients

	Univariate analysis		Multivariate analysis	
	*P*	Hazard ratio (95% CI)	*P*	Hazard ratio (95% CI)
Clinical stage				
Early stage (stage I-II)	< 0.001	2.061(1.513–2.816)	0.213	1.211(0.801–1.812)
Late stage (stage III-IV)				
T classification				
T1				
T2	< 0.001	1.396(1.178–1.715)	0.306	1.085(0.816–1.355)
T3				
T4				
N classification				
N0	< 0.001	1.782(1.370–2.152)	0.005	1.515(1.257–1.916)
N1				
M classification				
No	< 0.001	1.688(1.308–2.017)	0.016	1.558(1.219–1.889)
Yes				
Gankyrin expression				
Low expression	< 0.001	2.806(1.715–3.866)	< 0.001	2.311(1.518–3.102)
High expression				

### Gankyrin promotes aggressive phenotype in CRC cells *in vitro*

**Figure 2:**
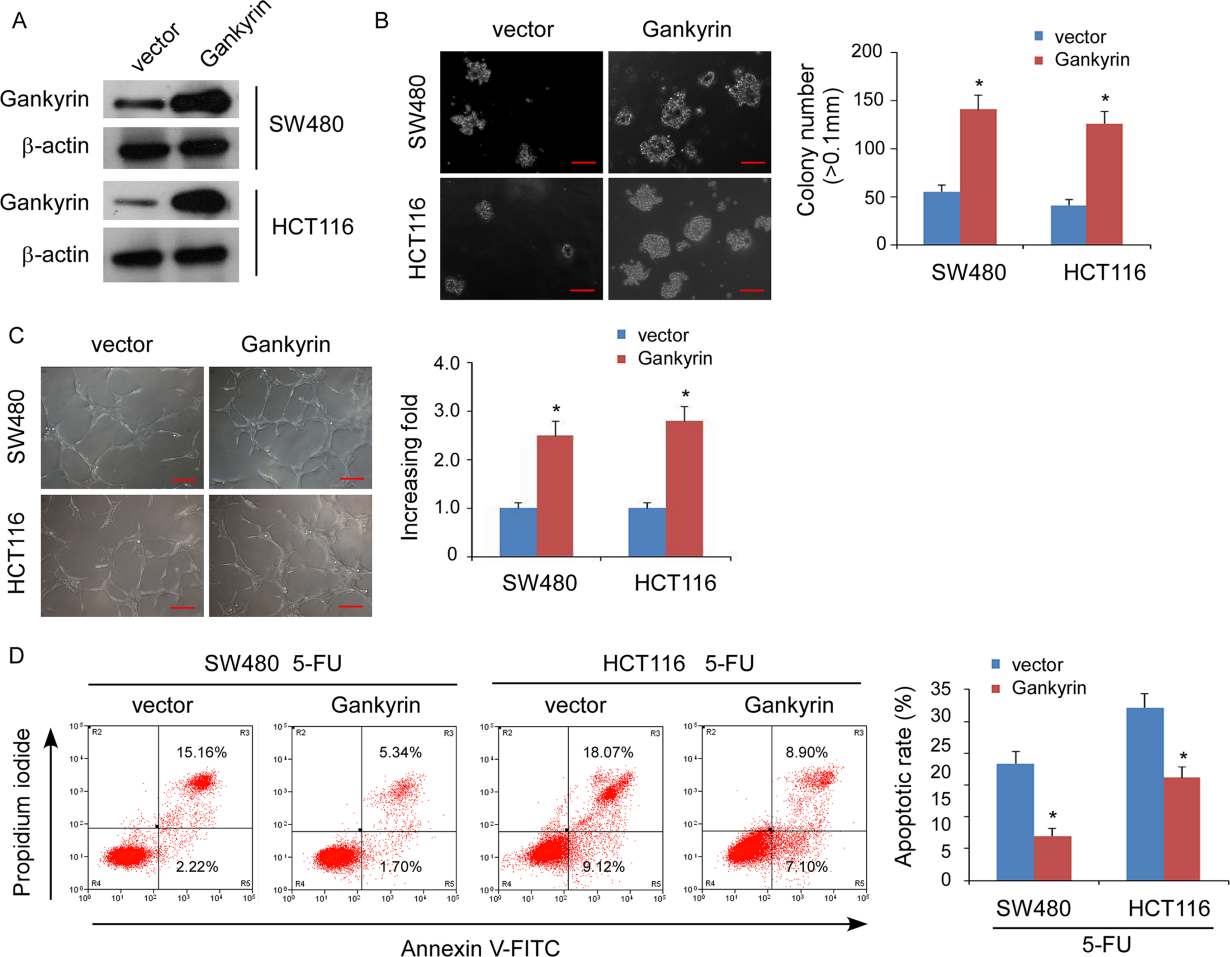
Overexpression of Gankyrin promotes aggressive phenotype of CRC cells *in vitro* (**A**) Overexpression of Gankyrin in SW480 and HCT116 cell lines analyzed by immunoblotting. β-actin served as the loading control. (**B**) Representative micrographs (left panel) and quantification (right panel) of colonies determined by anchorage-independent growth assays. Scale bars, 100 mm. Colonies > 0.1 mm in diameter were scored. (**C**) Representative images (left) and quantification (right) of tubule formation by HUVECs cultured on matrigel-coated plates with conditioned medium collected from the indicated CRC cells. Scale bars: 200 μm. (**D**) Annexin V-FITC/PI staining of the indicated cells after treatment with 5-fluorouracil (5-FU; 10 mM) for 36 h. Each bar represents the mean ± Standard Deviation (SD) of three independent experiments; *P < 0.05.

**Figure 3:**
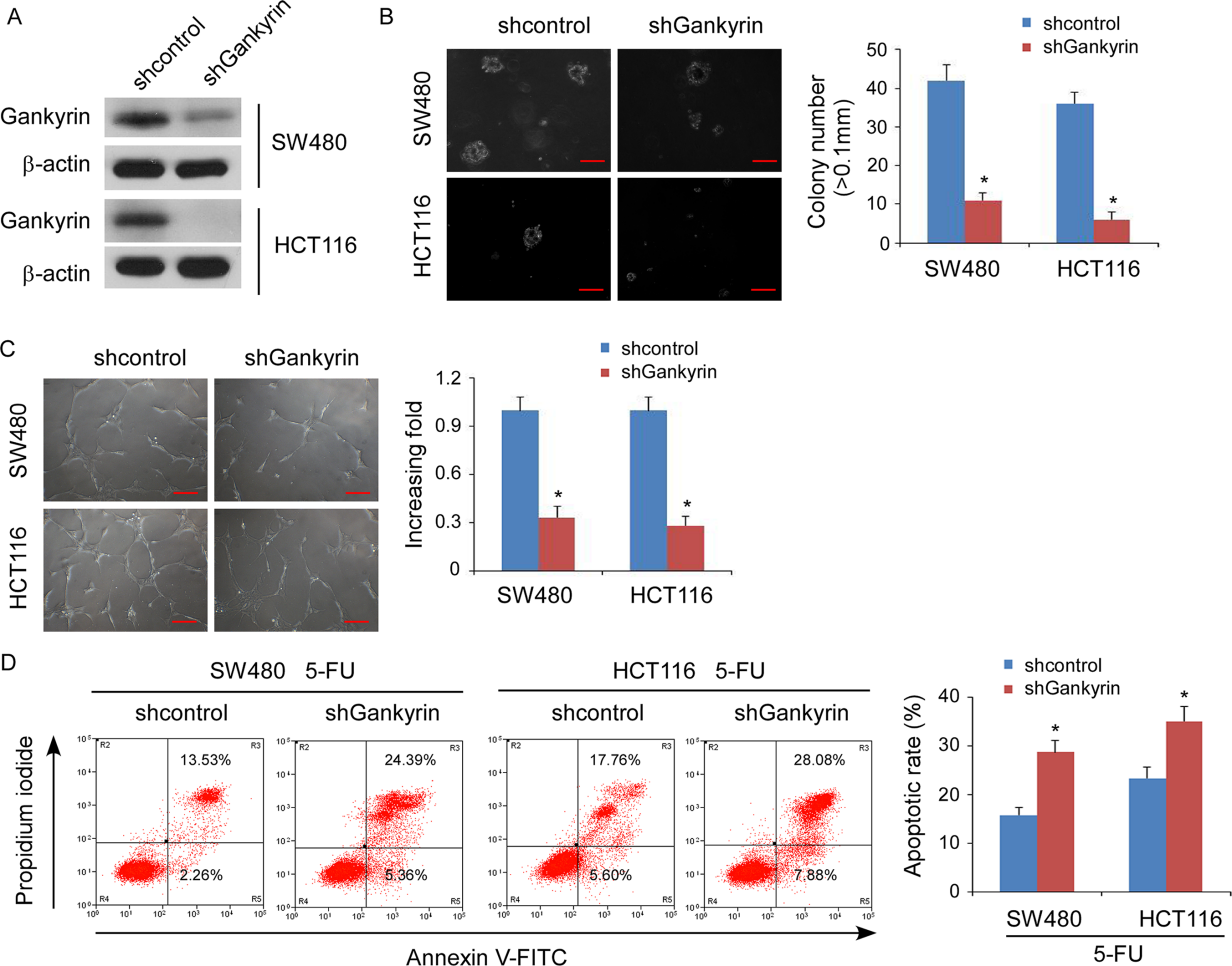
Knockdown of Gankyrin inhibits aggressive phenotype of CRC cells *in vitro* (**A**) Confirmation of the knockdown of Gankyrin by shRNA (#1) in SW480 and HCT116 cell lines. β-actin served as the loading control. (**B**) Representative micrographs (left panel) and quantification (right panel) of colonies determined by anchorage-independent growth assays. Scale bars, 100 mm. Colonies > 0.1 mm in diameter were scored. (**C**) Representative images (left) and quantification (right) of tubule formation by HUVECs cultured on matrigel-coated plates with conditioned medium collected from the indicated CRC cells. Scale bars: 200 μm. (**D**) Annexin V-FITC/PI staining of the indicated cells after treatment with 5-fluorouracil (5-FU; 10 mM) for 36 h. Each bar represents the mean ± SD of three independent experiments; *P < 0.05.

To investigate the biological function of Gankyrin during the pathogenesis of CRC, we established stable Gankyrin-expressing CRC cells using the cell lines SW480 and HCT116 (Figure 2A). Strikingly, overexpression of Gankyrin strongly enhanced the ability of the anchorage-independent growth abilities in CRC cells (Figure 2B), and induced tubule formation by human umbilical vein endothelial cells (HUVECs) (Figure 2C). Moreover, overexpression of Gankyrin conferred resistance to 5-fluorouracil-induced apoptosis in CRC cells (Figure 2D). Conversely, knockdown of Gankyrin significantly reduced the abilities of the invasion of CRC cells, and the tubule formation of HUVECs, and sensitized the CRC cells to 5-fluorouracil *in vitro* (Figure 3A–3D). Taken together, these results suggest that Gankyrin plays an important role in promoting the aggressive behavior of CRC cells.

### Gankyrin increases the motility and invasiveness of CRC cells *in vitro*

**Figure 4:**
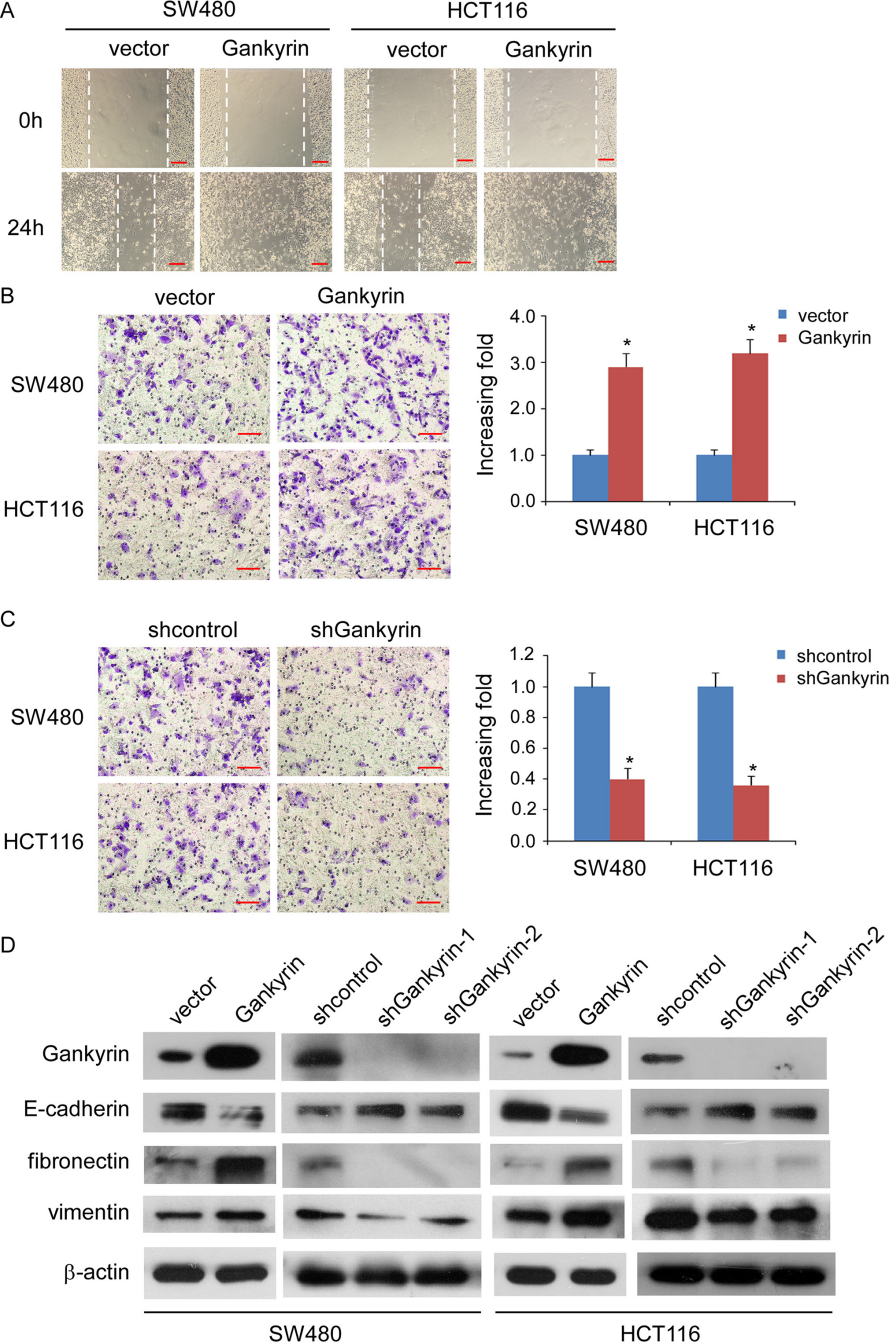
Gankyrin promotes invasiveness and EMT in CRC cells (**A**) Representative micrographs of wound healing assay of the indicated cells. Wound closures were photographed at 0 and 24 hours after wounding. Scale bars: 100 μm. (**B** and **C**) Representative micrographs of the transwell matrix penetration assay showing the invasiveness of Gankyrin-overexpressing cells (B) and Gankyrin-knockdown cells (C) compared with controls. Scale bars: 50 μm; Each bar represent the mean ± SD from three independent experiments, *P < 0.05. (**D**) Expression of epithelial cell and mesenchymal cell markers in indicated cells were examined by immunoblotting analysis. β-actin was used as a loading control.

Wound migration assays showed that upregulation of Gankyrin significantly increased the migratory speed of SW480 and HCT116 cells, whereas knockdown of Gankyrin abrogated the aforementioned the effect (Figure 4A, Supplementary Figure S2) Furthermore, the transwell matrix penetration assay showed that Gankyrin-overexpressing CRC cells enhanced the invasive capacity, whereas knockdown of Gankyrin inhibited the invasive capacity (Figure 4B, 4C). Moreover, we found that epithelial markers of E-cadherin was drastically downregulated, but mesenchymal markers such as vimentin and fibronectin were dramatically upregulated in Gankyrin-transduced cells, inversely, epithelial markers of E-cadherin was drastically increased, but mesenchymal markers of vimentin and fibronectin were dramatically decreased in Gankyrin-knockdown cells (Figure 4D). These results suggest that Gankyrin promotes motility and invasiveness in CRC cells.

### Gankyrin sustains PI3K/GSK-3β/β-catenin signal activation in CRC

**Figure 5:**
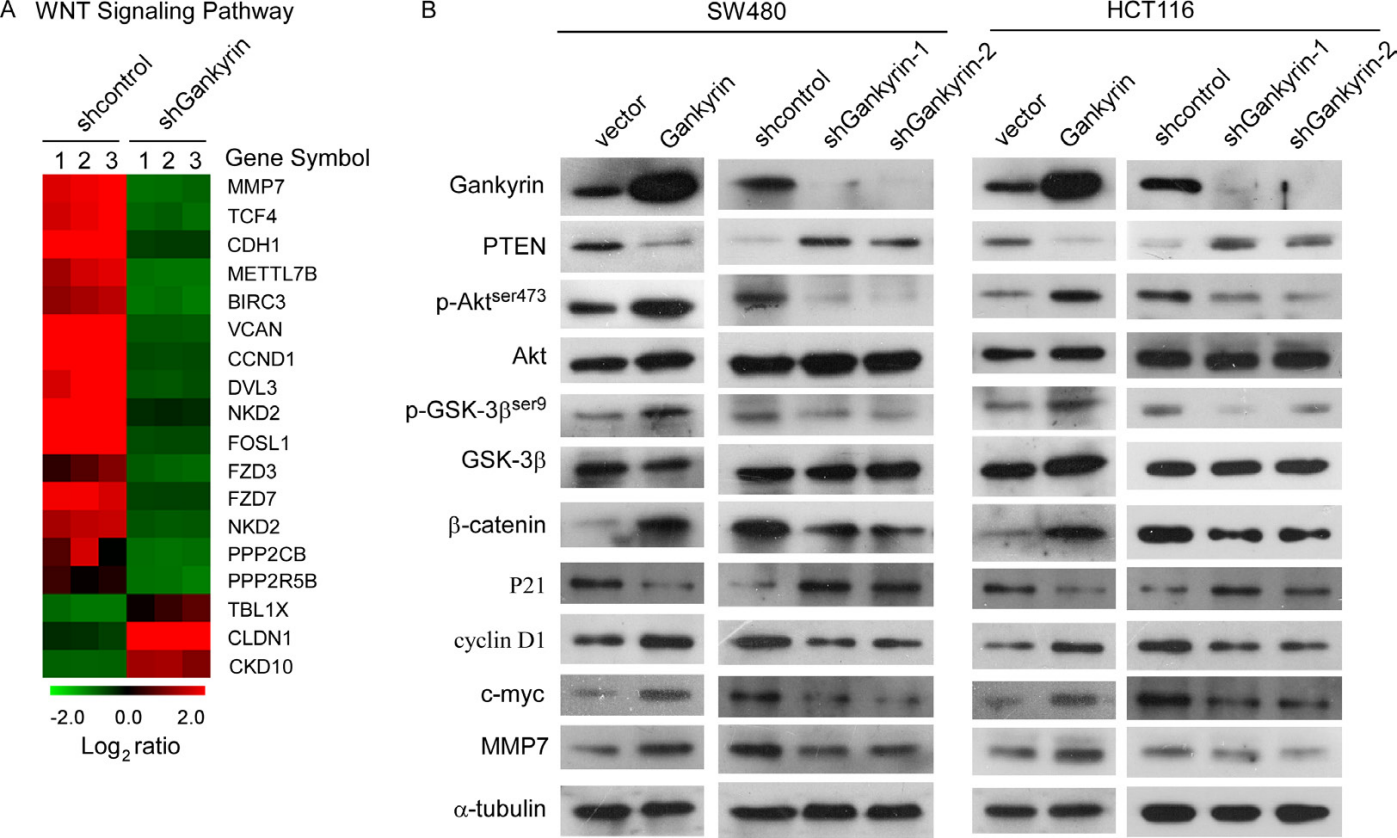
Gankyrin sustains PI3K/GSK-3β/β-catenin signal activation in CRC (**A**) Gene array analysis demonstrating an apparent overlap between WNT signaling pathway-dependent gene expression and Gankyrin-regulated gene expression. The pseudocolour represents the intensity scale for Gankyrin shRNA versus control shRNA, calculated by log_2_ transformation. (**B**) Western blotting analysis of the expression levels of the indicated proteins including PI3K/Akt and Wnt/β-catenin signaling pathway in the indicated cells, α-tubulin served as the loading control.

By analyzing published Gene expression profiles obtained from Gankyrin shRNA versus control shRNA (NCBI/GEO/GSE44029), we found that knockdown of Gankyrin significantly reduced mRNA expression levels of numerous well-characterized WNT downstream genes (Figure 5A), suggesting that Gankyrin may contribute to activation of WNT signaling pathway. Moreover, existing evidence demonstrated that Gankyrin played a central role in activating PI3K/Akt signaling pathway [24]. Furthermore, we investigated whether Gankyrin was involved in regulation of the PI3K/GSK-3β/β-catenin signal in CRC. As shown in Figure 5B, western blotting analysis demonstrated that the phosphorylation of Akt and GSK-3β, and β-catenin, and well-characterized WNT downstream genes such as cyclinD1, c-myc and MMP-7, were upregulated in Gankyrin-overexpressing cells and downregulated in Gankyrin-knockdown cells, nevertheless, overexpression of Gankyrin significantly reduced and knockdown of Gankyrin increased the expression of PTEN and P21, which demonstrating that Gankyrin contributed to activation of PI3K/GSK-3β/β-catenin signal.

### Gankyrin activates GSK-3β/β-catenin signal dependent on Akt activation

**Figure 6:**
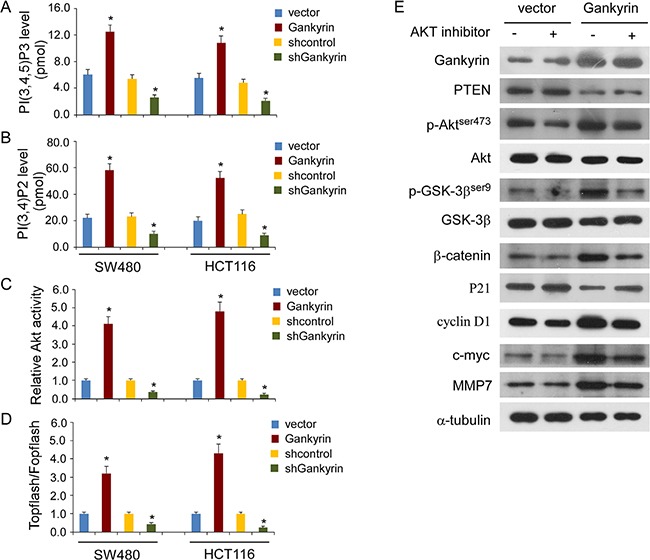
Gankyrin activates GSK-3β/β-catenin signal dependent on Akt activation (**A** and **B**) Detection of PI(3,4,5)P3 level (A) and PI(3,4)P2 level (B) in Gankyrin-transduced cells or Gankyrin-inhibited cells compared with controls. (**C**) Relative Akt activity in Gankyrin-overexpression or Gankyrin-knockdown CRC cells compared with controls. (**D**) Indicated cells transfected with TOPflash or FOPflash and Renilla pRL-TK plasmids were subjected to dual-luciferase assays. Reporter activity detected was normalized by Renilla luciferase activity. Each bar represents the mean ± SD of three independent experiments; *P < 0.05. (**E**) Western blotting analysis of the expression levels of the indicated proteins in indicated cells with or without treatment with the Akt inhibitor (MK-2206), α-tubulin served as the loading control.

To investigate whether the promoting effects of Gankyrin on PI3K/Akt signaling are associated with phosphoinositide metabolism, as shown in Figure 6A and 6B, Gankyrin overexpression significantly increased the levels of PI (3,4,5) P3 and PI (3,4) P2 in CRC cells, whereas knockdown of Gankyrin abrogated the aforementioned the effect. Consistently, the Akt activity was significantly induced in Gankyrin-overexpressing cells but inhibited in Gankyrin-knockdown cells (Figure 6C). Meanwhile, Gankyrin overexpression markedly increased the transcriptional activation of β-catenin in CRC cells, as determined by β-catenin reporter assay, whereas Gankyrin-inhibited cells had a lower transcriptional activation of β-catenin (Figure 6D). Next, we investigated whether activation of Akt signaling was required for Gankyrin-promoted transcriptional activation of β-catenin signal in CRC cells. As shown in Figure 6E, disruption of Akt signaling by treatment with Akt inhibitor abrogated the ability of Gankyrin-overexpressing to upregulation of the phosphorylation of Akt and GSK-3β, and β-catenin, and the expression of WNT downstream genes including cyclinD1, c-myc and MMP-7, and downregulation of the expression of PTEN and P21. These data indicated that Gankyrin activates GSK-3β/β-catenin signal dependent on Akt activation.

### Gankyrin knockdown impairs the metastasis capability of CRC cells to liver *in vivo*

**Figure 7:**
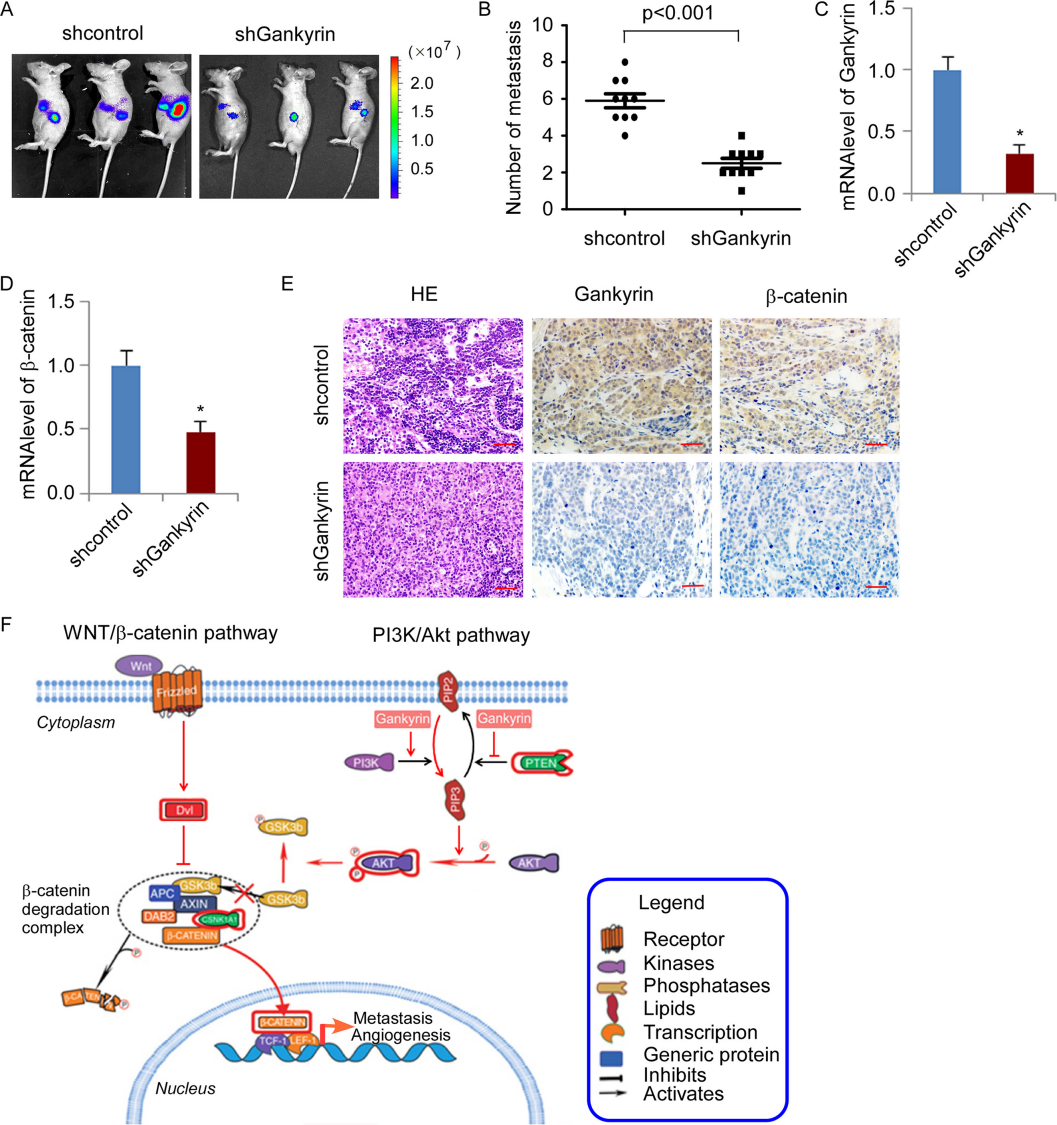
Knockdown of Gankyrin inhibited the metastasis capability of CRC cells to liver *in vivo* (**A**) Intrasplenic transplantation of HCT116/shGankyrin and HCT116/shcontrol cells expressing luciferase in BALB/c mice. The mice were staged for 28 days to allow the development of distant metastases. Bioluminescence images were acquired on whole animal. (**B**) Number of metastatic foci in liver was counted. (C and D) Q-PCR analysis of Gankyrin (**C**) and β-catenin (**D**) mRNA expression. Each bar represents the mean ± SD; *P < 0.05. (**E**) Hematoxylin/eosin staining and IHC staining of Gankyrin and β-catenin expression in enucleated tumors derived from liver. Scale bars, 50 μm. (**F**) Illustrative model showing the proposed mechanism by which Gankyrin promotes angiogenesis and metastasis in CRC via sustaining PI3K/GSK-3β/β-catenin signal activation.

Next, we investigated the effect of Gankyrin knockdown on colorectal cancer liver metastases using spleen-hepatic metastasis model *in vivo*. 5 × 10^6^ HCT116/shGankyrin and HCT116/shControl cells were injected into the spleen of BALB/c-nude mice, respectively. Bioluminescence imaging was used to visualize the metastasis in liver at day 28, showing Gankyrin knockdown significantly impaired hepatic metastasis (Figure 7A). Moreover, the number of liver surface metastatic foci was counted, showing that mice with injection of HCT116/shGankyrin cells dramatically decreased hepatic metastasis (*P* < 0.001, Figure 7B). Moreover, Gankyrin and β-catenin mRNA and protein expression were significantly reduced in enucleated tumors derived from HCT116 cells stably transfected with shGankyrin compared with control shRNA group, respectively (Figure 7C–7E). Collectively, these results further support the notion that Gankyrin overexpression promotes transcriptional activation of β-catenin by activating PI3K/Akt signalling, leading to CRC aggressiveness and progression (Figure 7F).

## DISCUSSION

Recent studies linked aberrantly activating Wnt/β-catenin signaling with the development and progression of CRC [2, 29, 30], including with CRC metastasis [31, 32]. Abnormal activation of the wingless related MMTV integration site (Wnt)/β-catenin pathway by mutations is responsible for the initiation of more than 90% of colon cancers [33]. Notably, our current data show that Gankyrin was upregulated in CRCs, which induced CRC cells proliferation, angiogenesis, chemoresistance and metastasis, by activating PI3K/GSK-3β/β-catenin signal, concordantly, promotion of CRC aggressiveness *in vitro* and *in vivo*. These findings uncover a novel mechanism for Gankyrin regulating the cross-talk among PI3K/Akt and Wnt/β-catenin signal in CRCs, and may represent a new target for clinical intervention in human CRCs.

Phosphoinositide 3-kinases (PI3Ks) belong to a conserved family of lipid kinases that phosphorylate the 3’-hydroxyl group of phosphoinositides. The most well-characterized product of this reaction is phosphatidylinositol-3,4,5-trisphosphate or PIP3, a critical second messenger that recruits AKT for activation of growth, proliferation and survival signaling [34–37]. Herein, we analyzed the published microarray analysis (NCBI/GEO/GSE44029) and found that genes in PI3K/Akt signaling pathway were down-regulated in Gankyrin knockdown cells, suggesting that Gankyrin governs this pathway in fulfilling its cellular function. Consistently, our results show that Gankyrin is substantially overexpressed in CRC and sustains PI3K/Akt signalling through enhancing PIP3 and PIP2 levels, moreover directly suppressing phosphoinositide phosphatase of PTEN expression. Earlier study has shown that PI3K/Akt pathway positively controlled Gankyrin expression, which increase resistance to DNA damage-induced apoptosis [38]. Therefore, the oncoprotein Gankyrin establishes a positive feedback loop in PI3K/Akt signaling.

Intriguingly, we found numerous β-catenin/TCF target genes varied expression levels concordant with Gankyrin knockdown (Figure 5A), thus, we asked whether there was a relationship between Gankyrin and wnt/β-catenin signal. As expected, our results demonstrated that overexpression of Gankyrin increased, whereas knockdown of Gankyrin suppressed, both the β-catenin protein expression and β-catenin/TCF transcriptional activity, consistently, including the levels of GSK-3β phosphorylation (the upstream of β-catenin) and the expression of cyclin D1, c-myc and MMP7 (the downstream targets of β-catenin), suggesting that Gankyrin govern GSK-3β/β-catenin signaling activation. Among those target genes, Cyclin D1 and c-Myc play a role in the G1-S checkpoint of cell cycle progression and is involved in cell migration and invasion [39, 40]. Therefore, Gankyrin can coordinately expressed with β-catenin, c-Myc and cyclin D1, further indicating that Gankyrin plays a role similar to that of c-Myc and cyclin D1 in CRC progression.

Previous studies have found that members of the lymphoid enhancer factor-1/T-cell factor (LEF-1/TCF) family interaction with β-catenin and their roles as the nuclear effectors of Wnt signaling [41, 42]. Dong *et al.* demonstrated that Gankyrin is regulated by β-catenin signaling, and Gankyrin positively regulates β-catenin/TCF transcriptional activity, however, they did not demonstrate the association between Gankyrin and GSK-3β or β-catenin [39]. In addition, the cross-talk between β-catenin signaling and PI3K/Akt pathway has been confirmed in a variety of cancers [43, 44]. Then, we speculate that whether Gankyrin regulates β-catenin signaling in a PI3K/Akt dependent manner. Expectedly, Akt inhibitor abrogated the ability of Gankyrin to activate GSK-3β/β-catenin signal, indicating that Gankyrin regulates GSK-3β/β-catenin signaling activation via a PI3K/Akt dependent mechanism in CRC. The present study demonstrates for the first time that PI3K/Akt plays pivotal roles in mediating Gankyrin signaling to β-catenin transcriptional activation.

Additionally, ELISA analysis verified that plasma concentrations of Gankyrin in CRC patients were elevated as compared with those of healthy individuals, suggesting that Gankyrin may be a noninvasive serological biomarker for early CRC diagnosis, which deserve to expand the sample size to study in clinical and evaluate the diagnostic efficacy of Gankyrin in early-stage CRC patients. Our clinical analysis also indicated that high expression of Gankyrin was associated with poorer overall survival in patients with CRC. Moreover, knockdown of Gankyrin inhibited the metastasis of CRC cells in a vivo spleen-hepatic metastasis model. Therefore, Gankyrin could potentially serve as a novel strategy to prevent CRC progression.

Consequently, our results clearly demonstrate the oncogenic potential of Gankyrin and suggest a unique molecular mechanism of Gankyrin in cell signaling transduction, by which sustains PI3K/GSK-3β/β-catenin signal activation. Therefore, these findings not only reveal a mechanism that promotes aggressiveness and progression in CRC, but also provide insight into novel molecular targets for antiangiogenic therapy in CRC.

## MATERIALS AND METHODS

### Patient information and tissue specimens

This study was conducted on a total of 217 paraffin-embedded CRC samples, which were histopathologically and clinically diagnosed at Guangzhou First People's Hospital from 2010 to 2016. No patients had received chemotherapy or radiotherapy before biopsy. For the use of these clinical materials for research purposes, prior patient consent and approval from the Institutional Research Ethics Committee were obtained. Pertinent follow-up information was available for all patients with CRC. Clinical information on the samples is summarized in Table 1. Ten CRC specimens and the matched adjacent noncancerous tissues were frozen and stored in liquid nitrogen until further use.

### Cell lines and cell culture

The colorectal cancer cell lines SW480 and HCT116 were purchased from the American Type Culture Collection (ATCC, Manassas, VA, USA) and cultured in 1640 medium (Invitrogen, Carlsbad, CA, USA) supplemented with 10% fetal bovine serum (Invitrogen), 100 U/ml penicillin and 100 μg/ml streptomycin (Invitrogen) in a humidified atmosphere of 5% CO_2_.

### Plasmids, virus constructs and retroviral infection of target cells

Human Gankyrin was amplified by PCR and cloned into the pSin-EF2 vector. Knockout of endogenous Gankyrin was performed by cloning two short hairpin RNA (shRNA) oligonucleotides into the pSuper-retro-puro vector to generate pSuper-retro- Gankyrin-shRNA(s). ShRNA targeting human Gankyrin sequence were: sense, 5′-GCTCAAGTGAATGCTGTCAAT-3′ (shRNA-1); and sense, 5′-CAAGGGTAACTTGAAGATGAT-3′ (shRNA-2). The shRNA targeting mouse Gankyrin sequence is: 5′-GCCATACAGAAATTGTTGAAT-3′. The scrambled derivative 5′-AAGCCAGAGACGTTACG TA-3′. Stable cell lines expressing Gankyrin and Gankyrin-shRNA were generated by retroviral infection using HEK293T cells. Viral infections were done serially, and stable cell lines expressing as previously described [45], and selected with 0.5 μg/ml puromycin for 10 days. The reporter plasmids containing wild-type (CCTTTGATC; TOPflash) or mutated (CCTTTGGCC; FOPflash) TCF/LEF DNA binding sites were provided kindly by Professor Jun Li (Sun Yat-Sen University, Guangzhou, China)

### Cell treatments

Akt inhibitor MK-2206 (1 mM; Selleck Chemicals, Houston, TX, USA), and 5-fluorouracil (10 mM; Sigma, St. Louis, MO, USA) was dissolved in dimethyl sulphoxide.

### RNA extraction and real-time quantitative PCR

As described previously [46], the PCR primer sequences of Gankyrin are: forward sequence: 5′-CAT CCAAGACACTGAGGGTAAC-3′, reverse sequence: 5′-AATACTTGCTCCTTGGGACAC-3′. The primer sequences of β-catenin are: forward sequence: 5′-TTGAA AATCCAGCGTGGAC-3′; reverse sequence: 5′-CGAGT CATTGCATACTGTC-3′. The primer sequences of GAPDH are: forward sequence: 5′-CTCCTCTGACTTCAA CAGCGAC-3′, reverse sequence: 5′-CCTGTTGCTGTAG CCAAATTCG-3′. In addition, the primers for the detection were designed and synthesized by Guangzhou RiboBio (RiboBio, China). Expression data were normalized to the geometric mean of housekeeping gene GAPDH to control the variability in expression levels. All experiments were performed in triplicate.

### Bioinformatics analysis of TCGA database

The mRNA expression profiling datasets have been deposited in THE CANCER GENOME ATLAS (http://cancergenome.nih.gov/) under colon and rectum adenocarcinoma data project, until to December 31, 2014. Genes that were altered by Limma moderated *t*-test at a *P*-value less than 0.05 between primary CRC tissues and normal colorectal tissues were considered significant for further analysis. Bioinformatics analysis and visualisation of microarray data were performed with MeV 4.6 (www.tm4.org/).

### Immunohistochemistry staining and evaluation

IHC analysis was performed in 217 clinical CRC tissue sections as previously described [47]. The working concentrations of primary antibody for the detection of Gankyrin (Santa Cruz Biotechnology, Santa Cruz, CA, USA) and β-catenin (Sigma, St. Louis, MO, USA) were 1:100 and 1:50, respectively. The sections were reviewed and scored independently by two observers based on both the proportion of positively stained tumor cells and the intensity of staining. The proportion of positive tumor cells was scored as follows: 0, no positive tumor cells; 1, < 10%; 2, 10%–35%; 3, 35%–75%; 4, > 75%. The intensity of staining was graded according to the following criteria: 1, no staining; 2, weak staining (light yellow); 3, moderate staining (yellow–brown); 4, strong staining (brown). The degree of Gankyrin immunostaining was defined as the proportion score multiplied by the staining intensity score. Using this method of assessment, the expression of Gankyrin was scored as 0, 2, 3, 4, 6, 8, 9, 12 and 16. Cut-off values were chosen on the basis of a measure of heterogeneity with the log-rank test statistical analysis with respect to overall survival. SI ≥ 8 was defined as high Gankyrin expression and SI < 8 was defined as low Gankyrin expression.

### Western blotting analysis

Western blotting was performed as previously described [48], using anti-Gankyrin (1:1000), anti-vimentin(1:1000), anti-PTEN(1:1000), anti-P21(1:1000), anti-cyclin D1(1:1000), anti-c-myc(1:1000), anti-MMP7 (1:1000) antibodies (Santa Cruz Biotech., Santa Cruz, CA), anti-E-cadherin(1:5000), anti-fibronectin (1:5000)antibodies (BD Biosciences, San Jose, CA), anti-p-Akt^ser473^(1:1000), anti-Akt (1:2000), anti-p-GSK-3b^ser9^ (1:1000), anti-GSK-3b (1:1000), anti-β-catenin (1:1000) antibodies (Cell Signaling, Danvers, MA). The membranes were stripped and re-probed with anti-α-tubulin (1:3000) antibody or anti-β-actin (1:5000) (Cell Signaling) as a loading control. The antibody concentration was according to the recommendation of respective specification.

### Enzyme-linked immunosorbent assay (ELISA)

Plasma samples were collected from a part of CRC patients (*n* = 81) and 81 age-matched individuals without CRC, collected from the Department of General Surgery, Guangzhou First People's Hospital. Gankyrin plasma levels were measured by quantitative ELISA (XINYU, ShangHai, China) according to the manufacturer's instructions. Briefly, the plasma was added to a well coated with Gankyrin polyclonal antibody and incubated with biotinylated monoclonal anti-Gankyrin antibody at room temperature for 2 h. Horseradish peroxidase was used to catalyze the color development, and terminated with stop solution. Absorbance was measured at 450 nm. The protein concentrations were determined relative to standard samples.

### HUVEC tube formation assay

HUVEC tube formation assay was performed as previously described [49]. Briefly, 200 μl of pre-cooled Matrigel (BD Biosciences, San Jose, CA, USA) was transferred into each well of a 24-well plate and polymerized for 30 minutes at 37°C. HUVECs (3 × 10^4^) in 200 μl of conditioned medium were added to each well and incubated at 37°C, 5% CO_2_ for 12 hours. The capillary tube structure was photographed under a 100× bright-field microscope, and quantified by measuring the total length of the completed tubes. Each condition was assessed in triplicate.

### Annexin V-propidium iodide binding assay

Apoptotic cells were differentiated from viable or necrotic cells using Annexin V-FITC Apoptosis Detection Kit (KeyGEN Biotech Inc., Nanjing, China). According to the manufacturer's instructions, briefly, after treatment with 5-fluorouracil (5-FU) (Sigma, St. Louis, MO, USA)) for 24 hours, cells were harvested, washed in ice-cold PBS, resuspended in binding buffer and incubated propidium iodide (PI) and Annexin V-fluorescein isothiocyanate (FITC) for 15 min in the dark. The samples were washed and resuspended in PBS, and immediately analyzed by fluorescence-activated cell sorting (FACS) on a EPICS XL-MCL flow cytometer (Beckman Coulter, Brea, CA, USA).

### The anchorage-independent growth assay

Indicated cells (5×10^3^) were trypsinized and seeded on 2% Matrigel coated in 24-well plates, and medium was refreshed every other day. Cells forming a anchorage-independent growth (spheres) were photographed at 2-day intervals for 10 days.

### Luciferase reporter assay

Cells were seeded in triplicate in 24-well plates and allowed to settle for 24 hours. Indicated plasmids plus 1 ng pRL-TK Renilla plasmid were transfected into the cells using Lipofectamine 2000 Reagent (Invitrogen, Carlsbad, CA, USA). 48 hours after transfection, Dual-Luciferase Reporter Assay (Promega, Fitchburg, WI) was performed according to the manufacturer's instructions, as previously described [50].

### Transwell matrix penetration assay

Cells (2 × 10^4^) were plated on the top side of a polycarbonate Transwell filter (pre-coated with Matrigel) in the BioCoat™ Invasion Chambers (BD Biosciences, San Jose, CA) and incubated at 37°C for 24 hours. The cells remaining on the upper surface were removed with cotton swabs. Cells that had migrated to the lower membrane surface were fixed in 1% paraformaldehyde, stained with hematoxylin and counted under an optical microscope (Ten random 200×fields per well). Cell counts are expressed as the mean number of cells per field of view. Three independent experiments were performed and the data are presented as mean ± standard deviation (SD).

### Akt activity assay

To measure kinase activities of CRC cells, Akt was precipitated by a specific anti-Akt antibody. The immune complexes were then incubated with a biotinylated peptide substrate that became phosphorylated in the presence of activated Akt. The phosphorylated substrates, which reflected the activity of Akt kinase in the extract, afterwards, quantified with the K-LISA Akt Activity Kit (Calbiochem, Darmstadt, Germany) that comprises a primary antibody recognizing the phosphorylated substrate peptides.

### Analysis of phosphoinositide levels

Phosphoinositides were extracted from cells and subjected to enzyme-linked immunosorbent assay (ELISA) assay using a PI(3,4,5)P3 or PI(3,4)P2 mass ELISA kit (Echelon, Salt Lake City, UT), performed as previously described [51]. Briefly, lipids were extracted with 2.25 ml of MeOH, CHCl3, 12 M HCl (80:40:1) for 15 min at room temperature and partitioned by centrifugation after the addition of 0.75 ml of CHCl3 and 1.35 ml of 0.1 M HCl. The lower phase was vacuum-dried and dissolved in appropriate buffers. Phosphoinositide samples and standards were then applied for the ELISA assay according to the supplier's instructions. The reaction was terminated by adding stop solution (0.5 M H_2_SO_4_), and the absorbance was measured at 450 nm. All experiments were performed in triplicate.

### Liver metastasis model

A liver metastasis model was established in male BALB/c-nude mice (8 weeks old). The mice were anesthesized and a small incision was made through the skin over the spleen after shaving. The spleen, visible through the abdominal wall, was grasped and a small incision was made over the tip. 5×10^6^ HCT116-Luc/shControl and HCT116-Luc/shGankyrin cells were injected through a 29-gauge needle into the parenchyma of the spleen. The spleen was removed 2 min later and the incision in the skin was closed. The animals were staged for 28 days to allow for the development of distant metastases. Animals were injected with 10 mg/kg D-luciferin (Xenogen, Alameda, CA, USA) in PBS intraperitoneally and anesthetized by isofluorane using the XGI-8 gas anesthesia system (Xenogen). Bioluminescence images were acquired by using the IVIS Imaging System (Xenogen) 10–15 min after injection. After animals were sacrificed, tumors and metastasis target organs were removed. All experiments were performed in accordance with the relevant guidelines and approved by our institute.

### Statistical analysis

All statistical analyses were carried out using SPSS13.0 statistical software (SPSS, Chicago, IL, USA). The chi-square test was used to analyze the relationship between Gankyrin expression and clinicopathological characteristics. Survival curves were plotted by the Kaplan-Meier method and compared using the log-rank test. Survival data were evaluated by univariate and multivariate Cox regression analyses. Multivariate models were constructed sequentially using the survival state, then adding Clinical stage, T classification, N classification, M classification, Gankyrin expression, respectively, in addition, other parameters exclusion by collinearity. Comparisons between groups for statistical significance were carried out with a 2-tailed paired Student's *t*-test. Statistical significance was defined as *P* < 0.05.

## SUPPLEMENTARY MATERIALS


