# Supplementary material for: Gankyrin sustains PI3K/GSK-3β/β-catenin signal activation and promotes colorectal cancer aggressiveness and progression
Source: Oncotarget. 2016 Nov 8;7(49):81156–71. doi: 10.18632/oncotarget.13215 (PMC5348383; doi:10.18632/oncotarget.13215)
Supplement: Supplementary file 1 [file oncotarget-07-81156-s001.pdf]

Gankyrin sustains PI3K/GSK-3β/β-catenin signal activation and promotes colorectal cancer aggressiveness and progression

Supplementary Materials

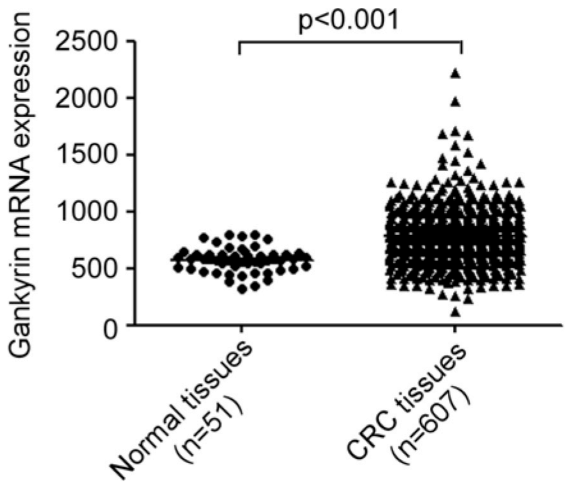

Supplementary Figure S1: The mRNA levels of Gankyrin was identified to be significantly upregulated in CRC tissues compared with normal tissues.

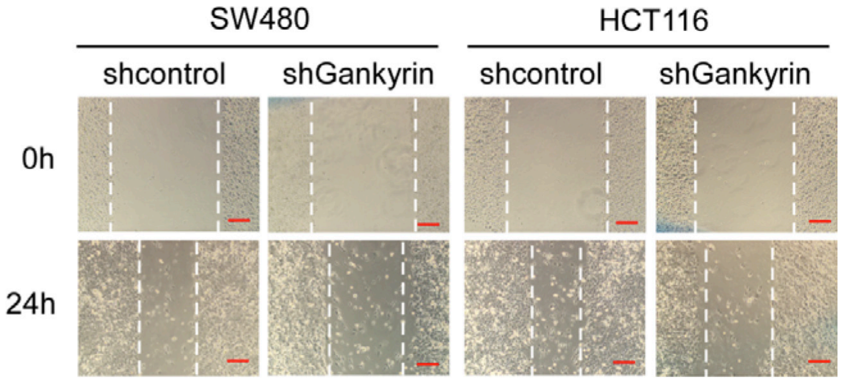

Supplementary Figure S2: Representative micrographs of wound healing assay of the indicated cells. Wound closures were photographed at 0 and 24 hours after wounding. Scale bars: 100 µm.
